# Deep Brain Stimulation of the Anterior Nucleus of the Thalamus in Drug-Resistant Epilepsy in the MORE Multicenter Patient Registry

**DOI:** 10.1212/WNL.0000000000206887

**Published:** 2023-05-02

**Authors:** Jukka Peltola, Albert J. Colon, José Pimentel, Volker A. Coenen, Antonio Gil-Nagel, Antonio Gonçalves Ferreira, Kai Lehtimäki, Philippe Ryvlin, Rod S. Taylor, Linda Ackermans, Jacqueline Ardesch, Carla Bentes, Magdalena Bosak, Jorge G. Burneo, Clara Chamadoira, Christian E. Elger, Loránd Erőss, Dániel Fabo, Howard Faulkner, Jacek Gawlowicz, Alireza Gharabaghi, Maurizio Iacoangeli, Jozsef Janszky, Soila Järvenpää, Elisabeth Kaufmann, Kuan H. Kho, Eva Kumlien, Helmut Laufs, Christian Lettieri, Paulo Linhares, Soheyl Noachtar, Andrew Parrent, Ekaterina Pataraia, Nikunj K. Patel, Ana Rita Peralta, Attila Rácz, Alexandre Rainha Campos, Ricardo Rego, Riccardo A. Ricciuti, Sabine Rona, Rob P.W. Rouhl, Andreas Schulze-Bonhage, Rick Schuurman, Mathieu Sprengers, Albert Sufianov, Yasin Temel, Tom Theys, Wim Van Paesschen, Dirk Van Roost, Rui Vaz, Kristl Vonck, Louis Wagner, Jack Zwemmer, Abdallah Abouihia, Thomas C. Brionne, Frans Gielen, Paul A.J.M. Boon, for The MORE Study Group

**Affiliations:** From the Department of Neurology (J. Peltola, S.J.), Tampere University and Tampere University Hospital, Finland; Academic Center for Epileptology Kempenhaeghe/MUMC+ (A.J.C., L.A., R.P.W.R., L.W.), Maastricht, the Netherlands; Department of Neurosciences and Mental Health (J. Pimentel), Hospital de Santa Maria, Centro Hospitalar Universitário Lisboa Norte, Portugal; Department of Stereotactic and Functional Neurosurgery (V.A.C.), Universitätsklinikum Freiburg, Germany; Neurology Department (A.G.-N.), Epilepsy Program, Hospital Ruber Internacional, Madrid, Spain; Department of Neurosurgery (A.G.F., A.R.C.), Hospital Santa Maria Centro Hospitalar Lisboa Norte, Portugal; Department of Neurosurgery (K.L.), Tampere University Hospital and Tampere University, Finland; Département des Neurosciences Cliniques (P.R.), centre hospitalier universitaire vaudois (CHUV), Lausanne, Switzerland; MRC/CSO Social and Public Health Sciences Unit & Robertson Centre for Biostatistics (R.S.T.), Institute of Health and Well Being, University of Glasgow; College of Medicine and Health (R.S.T.), University of Exeter, United Kingdom; Departments of Neurosurgery (L.A., Y.T.), Maastricht University Medical Center, the Netherlands; Stichting Epilepsie Instellingen Nederland (SEIN) (J.A.), Zwolle; Neurophysiology Monitoring Unit (C.B.), Department of Neurosciences and Mental Health, Hospital de Santa Maria, Centro Hospitalar Universitário Lisboa Norte; Faculdade de Medicina (C.B., A.R.P.), Universidade de Lisboa, Portugal; Department of Neurology (M.B.), Jagiellonian University Medical College, Faculty of Medicine, Krakow, Poland; Western University (J.G.B., A.P.), London, Ontario, Canada; Neurosurgery Department (C.C., P.L., R.V.), Centro Hospitalar Universitário de São João, Porto, Portugal; Department of Epileptology (C.E.E., A.R.), University Hospital Bonn, Germany; National Institute of Clinical Neuroscience (L.E.); Országos Idegtudományi Intézet/National Institute of Neurosciences (D.F.), Budapest, Hungary; North Bristol NHS Trust (H.F.), Bristol, United Kingdom; Wojewodzki Szpital Specjalistyczny w Lublinie (J.G.), Poland; Institute for Neuromodulation and Neurotechnology (A.G.), Department of Neurosurgery and Neurotechnology, University of Tübingen, Germany; Department of Neurosurgery (M.I.), Umberto I General University Hospital, Università Politecnica delle Marche, Ancona, Italy; Department of Neurology (J.J.), Medical School, University of Pécs, Hungary; Department of Neurology (E. Kaufmann), Ludwig Maximilians University, Munich, Germany; Medisch Spectrum Twente (MST) (K.H.K.), Enschede, the Netherlands; Department of Neuroscience (E. Kumlien), Uppsala University, Sweden; Klinik für Neurologie (H.L.), Universitätsklinikum Schleswig-Holstein, Campus Kiel, Christian-Albrechts-Universität zu Kiel, Germany; Neurology and Clinical Neurophysiology Unit (C.L.), Department of Neuroscience, “S. Maria della Misericordia” University Hospital, Udine, Italy; Klinikum der Universität München (S.N.), Großhadern Neurologische Klinik und Poliklinik, Germany; Medizinische Universität Wien (E.P.), Austria; Southmead Hospital (N.K.P.), North Bristol NHS Trust, United Kingdom; Laboratory of EEG/Sleep (A.R.P.), Department of Neurology, Department of Neurosciences and Mental Health, Hospital de Santa Maria, Centro Hospitalar Universitário Lisboa Norte, Portugal; Neurophysiology Unit (R.R.), Neurology Department, Centro Hospitalar Universitário de São João, Porto, Portugal; Department of Neurosurgery (R.A.R.), Viterbo Hospital, Italy; Epilepsy Unit (S.R.), Department of Neurosurgery and Neurotechnology, University of Tübingen, Germany; Department of Neurology (R.P.W.R.), Maastricht University Medical Centre+; School for Mental Health and Neurosciences (R.P.W.R.), Maastricht University, the Netherlands; University Hospital Freiburg (A.S.-B.), Germany; Amsterdam University Medical Center (R.S.), the Netherlands; Department of Neurology (M.S.), Ghent University Hospital, Ghent University, Belgium; Federal Centre of Neurosurgery (Tyumen) (A.S.), I.M. Sechenov First Moscow State Medical University, Moscow, Russia; UZ Leuven (T.T.); Department of Neurology (W.V.P.), UZ Leuven; Laboratory for Epilepsy Research (W.V.P.), KU Leuven; Department of Neurosurgery (D.V.R.), and Department of Neurology (K.V., P.A.J.M.B.), Ghent University Hospital, Ghent University, Belgium; Stichting Epilepsie Instellingen Nederland (SEIN) (J.Z.), Heemstede; Clinical Department (A.A., T.C.B.), Medtronic Internal Trading Sàrl, Tolochenaz, Switzerland; and Medtronic Bakken Research Center (F.G.), Maastricht, the Netherlands.

## Abstract

**Background and Objectives:**

The efficacy of deep brain stimulation of the anterior nucleus of the thalamus (ANT DBS) in patients with drug-resistant epilepsy (DRE) was demonstrated in the double-blind Stimulation of the Anterior Nucleus of the Thalamus for Epilepsy randomized controlled trial. The Medtronic Registry for Epilepsy (MORE) aims to understand the safety and longer-term effectiveness of ANT DBS therapy in routine clinical practice.

**Methods:**

MORE is an observational registry collecting prospective and retrospective clinical data. Participants were at least 18 years old, with focal DRE recruited across 25 centers from 13 countries. They were followed for at least 2 years in terms of seizure frequency (SF), responder rate (RR), health-related quality of life (Quality of Life in Epilepsy Inventory 31), depression, and safety outcomes.

**Results:**

Of the 191 patients recruited, 170 (mean [SD] age of 35.6 [10.7] years, 43% female) were implanted with DBS therapy and met all eligibility criteria. At baseline, 38% of patients reported cognitive impairment. The median monthly SF decreased by 33.1% from 15.8 at baseline to 8.8 at 2 years (*p* < 0.0001) with 32.3% RR. In the subgroup of 47 patients who completed 5 years of follow-up, the median monthly SF decreased by 55.1% from 16 at baseline to 7.9 at 5 years (*p* < 0.0001) with 53.2% RR. High-volume centers (>10 implantations) had 42.8% reduction in median monthly SF by 2 years in comparison with 25.8% in low-volume center. In patients with cognitive impairment, the reduction in median monthly SF was 26.0% by 2 years compared with 36.1% in patients without cognitive impairment. The most frequently reported adverse events were changes (e.g., increased frequency/severity) in seizure (16%), memory impairment (patient-reported complaint, 15%), depressive mood (patient-reported complaint, 13%), and epilepsy (12%). One definite sudden unexpected death in epilepsy case was reported.

**Discussion:**

The MORE registry supports the effectiveness and safety of ANT DBS therapy in a real-world setting in the 2 years following implantation.

**Classification of Evidence:**

This study provides Class IV evidence that ANT DBS reduces the frequency of seizures in patients with drug-resistant focal epilepsy.

**Trial Registration Information:**

MORE ClinicalTrials.gov Identifier: NCT01521754, first posted on January 31, 2012.

Of the 50 million people with epilepsy worldwide, one-third continue to have seizures despite appropriate antiseizure medications (ASMs).^1^ Many patients with drug-resistant epilepsy (DRE) cannot undergo resective epilepsy surgery due to poorly localized or multifocal onset^2^ or seizure origin within eloquent cortex. Depending on the underlying pathology, 26%–64% of patients continue to have seizures following epilepsy surgery.^3^ For all above patients with DRE, neuromodulation treatments are an option,^4^ including deep brain stimulation (DBS).

The efficacy of DBS of the anterior nucleus of the thalamus (ANT DBS) in patients with DRE was demonstrated in the Stimulation of the Anterior Nucleus of the Thalamus for Epilepsy (SANTE) pivotal trial^5^ and during 10 years of extended follow-up.^6^ After the SANTE trial was completed, questions remain regarding which patients are the best candidates. Several single-center studies provide additional support for the safety and efficacy of ANT DBS but are limited in sample size, making generalizability difficult.^7-21^ Consensus guidelines are based on a combination of the SANTE study, single-center studies, and expert opinion.^22^

To address these limitations and gain additional experience with ANT DBS, we designed a large multinational registry. The registry permitted patients with difficult-to-treat focal epilepsy and comorbidities to be assessed, with physicians electing to use DBS targeting approaches and leads not evaluated in the prior studies. The primary research questions in this study are as follows: to present (1) the clinical characteristics of the patients, (2) changes in seizure frequency (SF) and health-related quality of life (HRQOL), (3) adverse events (AEs) and (4) stimulation parameters of ANT DBS up to 24 months of follow-up.

## Methods

### Study Design

The Medtronic Registry for Epilepsy (MORE) registry is an open-label, observational, international study that collected both prospective and retrospective data to evaluate the long-term therapy effectiveness, safety, and performance of a neuromodulation system for ANT DBS for the treatment of DRE. The study was designed to characterize patients implanted with DBS, identify factors predicting response to the therapy, and explore potential trends affecting changes in the SF. Patient-reported outcomes, including HRQOL and cognitive performance, were also assessed. Sites were selected partly based on their documented previous expertise with resective surgery for DRE, with DBS in other indications such as movement disorders, and access to a patient pool of sufficient potential to recruit patients meeting inclusion criteria.

### Participants

Eligible participants were at least 18 years old with focal DRE as recorded in a seizure diary. The seizures were originally classified by each site according to the International League Against Epilepsy (ILAE) 1981 classification with the corresponding seizure types matching the ILAE 2017 classification^23^ presented in Table 1. In case of discordance between seizure type and epilepsy type, seizures were reassessed by the primary and last authors based on all clinical and EEG data available. To fulfill the inclusion criteria, at least 2 ASMs failed to benefit the patient. Exclusion criteria encompassed inaccessibility for follow-up or incomplete and/or unreliable seizure diaries based on the treating physician's judgment. Vagus nerve stimulation (VNS), if present, was recommended to be handled per instructions by the treating physician.

**Table 1:**
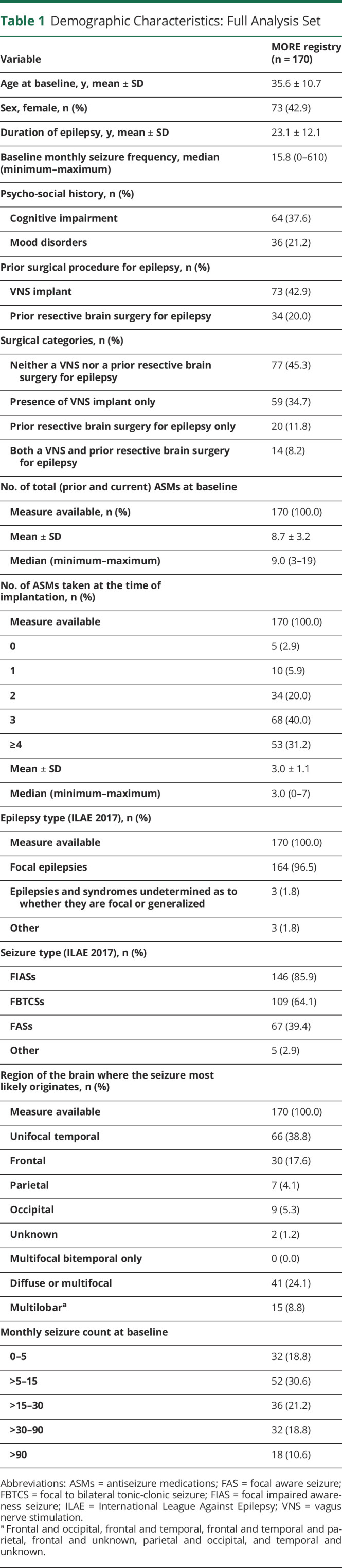
Demographic Characteristics: Full Analysis Set

Less than one-third of participants were enrolled a median of 12.6 months after implantation, with preenrollment data available for baseline and follow-up seizure frequencies, but the data were collected retrospectively. Patients did not have to be consecutive.

### Surgical and Clinical Procedure

The patients needed to have a minimum of 2 full consecutive calendar months of seizure diary before DBS surgery. At the baseline visit, seizure diary data and other inclusion and exclusion criteria were reviewed to determine whether patients remained eligible to continue participation in the study. The implant visit was then performed according to the physician's standard clinical practice. To ensure the use of CE-marked implantable devices, the protocol called for implant with Medtronic Model 3387 DBS leads (Medtronic PLC, Dublin, Ireland), connected to a dual channel Activa PC Neurostimulator (Medtronic) via extensions tunneled subcutaneously, according to the labeling. However, the final selection of the leads was performed by the DBS center. DBS electrodes were aimed to be implanted in the ANT bilaterally using a stereotactic technique. The implantation procedure was performed per local standard clinical practice. Lead positions were verified postoperatively with MRI and/or CT. The decision of a need for reimplantation was based on the clinical judgment of the operating center. Timing and dosage of stimulation, we well as medication adjustments were per each center's chosen clinical practice, but mainly driven by SANTE study advised practice.

Once enrolled, patients were followed at least for 24 months (to comply with the primary objective of the registry). An extension of the follow-up to 5 years was implemented in February 2016, that is, after some patients had already exited the original study, and was discontinued when all subjects reached 2 years, that is, before some subjects could reach 5 years. Follow-up visits took place according to clinical practice. Visits were grouped in intervals of 3, 6, and every 6 months thereafter. Adverse events and/or device events were reported per the Clinical investigation of medical devices for human subjects—good clinical practice (ISO 14155:2011), which calls for any change in frequency or severity of a symptom to be reported. All events (including worsening of epilepsy/seizure) were classified using the Medical Dictionary for Regulatory Activities version 22.0; an external adjudication committee reviewed all adverse events for potential relation to DBS devices, programming, or procedures. Medications were coded using World Health Organization Drug version 2b2016SEP.

### Standard Protocol Approvals, Registrations, and Patient Consents

The registry was conducted in accordance with the Declaration of Helsinki and regulations of the country in which the registry was conducted, including data protection laws. Ethics committee approval was obtained at each center, except in Poland, where ethics committee approval was not needed according to local laws and regulations. All participants granted ethics committee/institutional review board–approved and informed consent/data release consent. The trial was registered on January 31, 2012, at ClinicalTrials.gov (NCT01521754).

### Statistical Analysis Methods

Two analysis sets were used in this study: all patients set (APS) consisted of all enrolled patients and full analysis set (FAS) consisted of patients of the APS who met all eligibility criteria and were implanted with DBS therapy for epilepsy following the intent to treat (ITT) principle. APS was used for safety analysis and FAS for assessing the primary effectiveness objective, that is, the relative change in monthly SF at 2 years postimplant, and secondary effectiveness objectives including Quality of Life in Epilepsy 31-item questionnaire (QOLIE-31) and Beck Depression Inventory II (BDI-II) scores. No missing data imputation was used for the analyses of primary and secondary objectives. The patients were categorized into 3 groups based on the seizure reduction (SR) at 2 years of follow-up: responders (≥50% SR), improvers (50% > SR > 0%), and no-benefit patients (SR ≤0%).

Baseline and follow-up data were summarized using appropriate summary statistics. Although no formal hypothesis testing was defined for this study, *p* values were calculated to provide context for the relevance of the changes observed within paired data. Change from baseline was tested using the paired Student *t* test or nonparametric methods (e.g., Wilcoxon signed-rank test) as appropriate, and comparison between different groups was tested using the Student test or analysis of variance analysis or Kruskal-Wallis test for continuous variables and using the Fisher exact test for categorical variables. Statistical tests were examined for significance at the 5% level, with no adjustments for multiple comparison.

Safety data were summarized using descriptive statistics. In addition, adverse events were reported per study period; for each period, the available patient set was used. All analyses were performed using SAS software (version 9.4; SAS Institute, Cary, NC).

### Data Availability

Individual patient data cannot be made available under local law because we did not obtain patient approval for routinely sharing individual patient data outside the MORE study group, even in coded form. However, request for syntax files and output of statistical analyses will be evaluated by the MORE publication committee.

## Results

**Figure 1:**
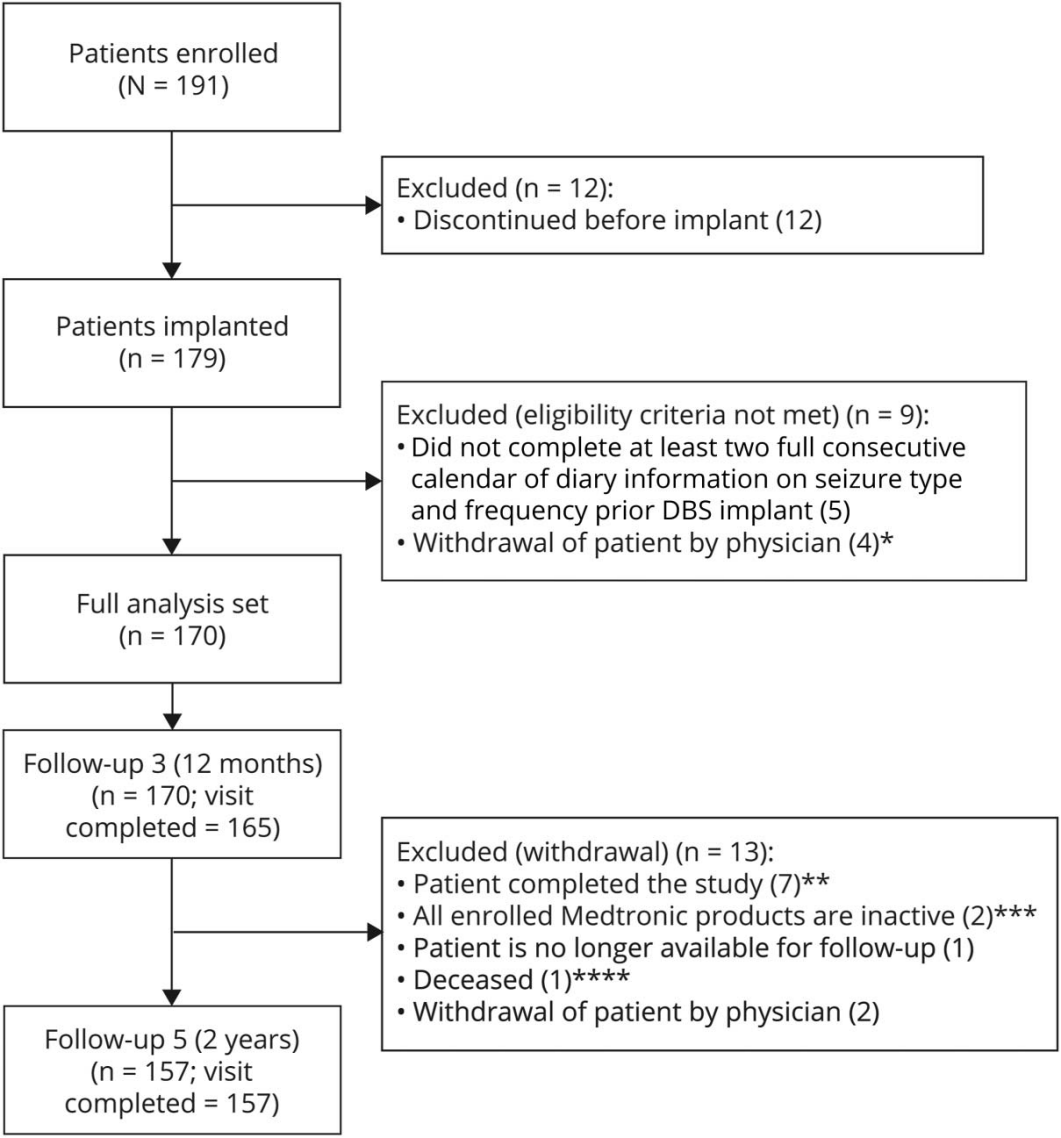
Patient Disposition: Full Analysis Set *Four patients were enrolled before the site was accredited to participate in the registry. **Seven patients completed and reported 2 years' visits before follow-up 5. ***Two patients explanted without replacement due to infection. ****Deceased was assessed by external advisory committee as not related to DBS therapy. DBS = deep brain stimulation.

Patient enrollment was from February 21, 2012, until April 30, 2017. The last data were collected on June 19, 2019. A total of 191 (APS) patients were recruited by 25 centers in 13 countries, with recruitment varying from 1 to 28 patients per site. Most of the 170 FAS subjects (157, 92.4%) completed follow-up visit 5 (scheduled around 2 years after implantation) for a median of 2 years of follow-up. Reasons for 13 patients withdrawn before follow-up visit 5 are given in Figure 1.

### Baseline Characteristics (FAS)

The mean (SD) age was 35.6 (10.7) years, and 43% of the patients were female. Nearly all (96%) patients had a diagnosis of localization-related epilepsy (1989 ILAE epilepsy syndrome classification), that is, focal epilepsy.^24^ Their clinical characteristics are reported in Table 1. The mean (SD) duration of epilepsy was 23.1 (12.1) years, and the median monthly SF at baseline was 15.8, with 32 patients with 5 or less seizures per month and 18 patients with more than 90 seizures per month. The majority of patients had focal impaired awareness seizures (FIASs; 86%) and focal to bilateral tonic-clonic seizures (FBTCSs; 64%). The median number of ASMs tried before or currently taken at baseline was 9. In 34 (20%) patients, at least 1 resective epilepsy surgery procedure had been performed before ANT DBS. A VNS system was previously implanted in 73 (43%) MORE patients. Among these, 28 (38%) had VNS explanted before DBS implant. Of the patients who still had VNS at the time of the DBS implant, 24 (53%) continued VNS therapy during the MORE data collection. At entry, 38% of patients reported cognitive impairment, and 21% experienced mood disorders (Table 1).

Nearly all (94%) patients received an Activa PC stimulator. The most frequently implanted bilateral lead model was the 3389 in 76% of patients (eTable 1, links.lww.com/WNL/C695).

### Outcomes

**Figure 2:**
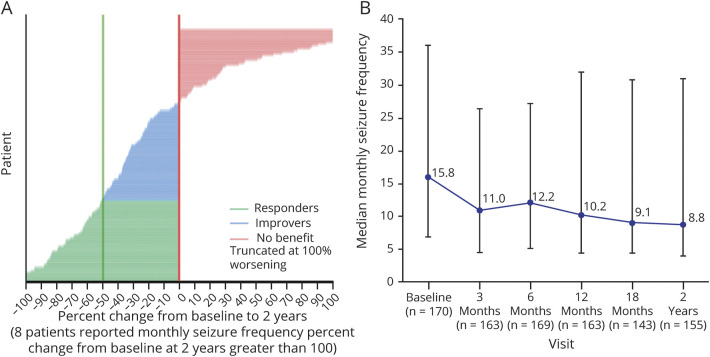
Seizure Frequency Percent Change From Baseline to 2 Years (A) Monthly seizure frequency percent change from baseline to 2 years—plot by patient: full analysis set. (B) Median monthly seizure frequency from baseline through 2 years: full analysis set. Green: responders. Blue: improvers. Pink: no benefit, truncated at 100% worsening. Five patients were seizure-free. The range of increase in median monthly seizure frequency in patients with more than 100% increase was 101%–6,519%.

**Figure 3:**
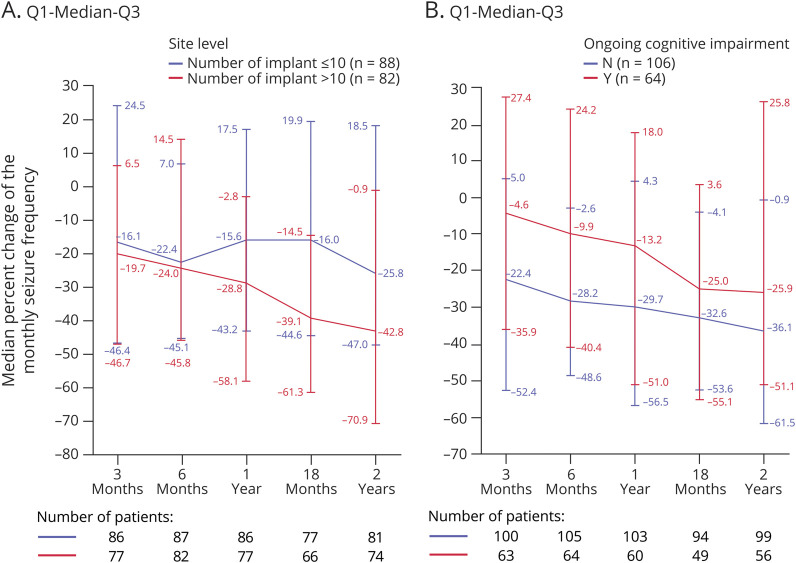
Unadjusted Median Monthly Seizure Frequency Change From Baseline to 2 Years: Full Analysis Set (A) By center implant group. (B) By cognitive impairment. Error bars represent the interquartile range. Differences (A) between center implant groups and (B) cognitive impairment groups are not statistically significantly different using a generalized linear mixed model.

The median monthly SF progressively decreased from 15.8 at baseline to 8.8 at 2 years of follow-up (Figure 2B) corresponding to a median reduction of 33.1% (*p* < 0.0001, eFigure 1, links.lww.com/WNL/C695). 32.3% of patients were responders, including 5 seizure-free patients at 2 years of follow-up (Figure 2A). The 24 patients who received both DBS and VNS achieved a similar median reduction of 33.1%. In the subgroup of patients (n = 47) who had completed an additional 3 years of follow-up, the median SR reported at 2 years was 43.3% and at 5 years was 55.1% (Table 2). The responder rate (RR) in this subgroup was 40.4% at 2 years and 53.2% at 5 years. Patients from 21 centers who had performed 10 or less ANT DBS (low-volume centers) showed a median monthly frequency reduction at 2 years of follow-up of 25.8% and those from 4 centers with more than 10 implantations (high-volume centers) a reduction of 42.8% (*p* = NS, Figure 3A). Patients with cognitive impairment at baseline experienced a median SR of 26.0% and those without cognitive impairment a reduction of 36.1% (*p* = NS, Figure 3B).

**Table 2:**
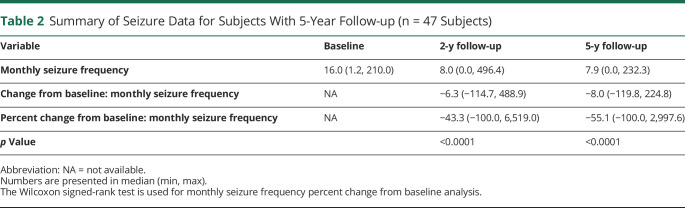
Summary of Seizure Data for Subjects With 5-Year Follow-up (n = 47 Subjects)

Although baseline characteristics of responders, improvers, and no-benefit groups appeared to differ (eTable 2A, links.lww.com/WNL/C695), most did not reach statistical significance, potentially due to the limited sample size of each group. The RRs of patients with frontal, temporal, or other seizure onset zones were around 30% (eTable 2B). However, taking all seizure types, the median SF change in temporal lobe seizures ranged from −27.9% at 1-year to 32.6% at 2-year follow-up; frontal seizures were also reduced (−18.4% to −34.7%), as well as seizures originating in other lobes (−17.1% to −24.8%; all *p* < 0.05 at each follow-up; eTable 2C). At 2-year follow-up, the RR was 43.2% in high-volume centers and 22.2% in low-volume centers (*p* < 0.05). In total, 50 of 155 subjects with 2-year data were responders, which included 32 (64%) from high-volume centers and 18 (36%) from low-volume centers. In eTable 4, the baseline characteristics of the patients are compared between the more experienced and less experienced centers, and there are no statistically significant differences for most of the baseline variables, except for a higher proportion of FIAS in the high-volume sites and a higher proportion of FBTCS in the low-volume centers.

We observed an overall 2-point mean improvement in QOLIE-31 (*p* < 0.05, eFigure 2, links.lww.com/WNL/C695), additionally about one-third of patients improved ≥5 points in the QOLIE-31 (*p* = 0.02, eFigure 3) at 2 years. These measures were only available for 78 patients.

No statistically significant change in the prevalence or severity of depression was observed (*p* > 0.05 for all visits, eFigure 4, links.lww.com/WNL/C695); BDI scoring was available for only 87 patients. Over the 2-year period, none of the stimulation parameters including amplitude, pulse width, stimulation frequency, or percentage of patients on intermittent cycling stimulation were significantly changed (eTable 5, links.lww.com/WNL/C695).

The most frequently reported adverse events were changes (e.g., increased frequency/severity) in seizure (16%), memory impairment (patient-reported complaint, 15%), depressive mood (patient-reported complaint, 13%), and epilepsy (12%). Thirty-nine percent of the memory impairment complaints and 44% of the depressive mood complaints were related to worsening of preexisting conditions per site assessment. Other adverse events included headache (7%), head injury (5%), irritability (5%), anxiety (5%), and cognitive impairment (5%, Table 3).

**Table 3:**
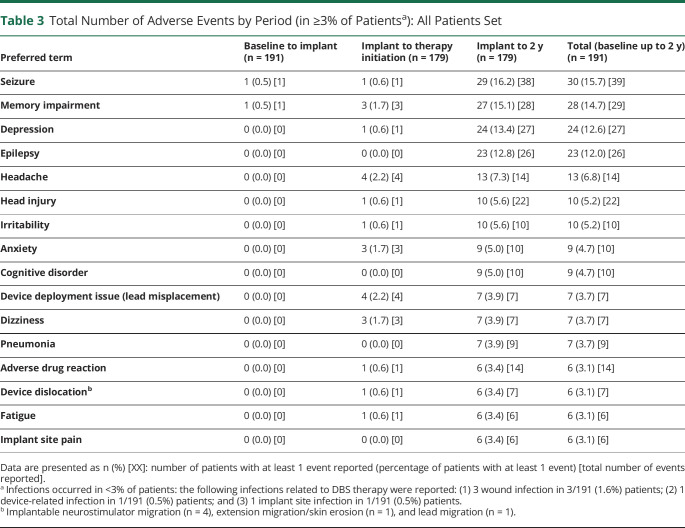
Total Number of Adverse Events by Period (in ≥3% of Patients^a^): All Patients Set

The subgroup of 24 patients with both DBS and VNS had a similar safety profile as the rest of the population (data not shown). A total of 160 serious AEs (SAEs) were reported in 83 subjects (43.5%) from baseline to 2-year follow-up. The most frequently reported SAE was seizure in 18 subjects (10.1%). In this study, SAEs reported as related to procedure, lead, neurostimulator, extension, other DBS component, or programming/stimulation were classified as DBS related. Overall, 59 DBS-related SAEs were reported in 42 subjects (23%) in the 2 years after implant. None of these SAEs led to discontinuation from the study. As with general SAEs, the most frequently reported DBS-related SAEs were seizure in 7 subjects (3.9%). DBS-related SAEs were connected to the procedure (n = 24) or to stimulation/programming (n = 21), whereas 11 (6.1%) were linked to implanted system components. A total of 11 subjects (6.1%) had lead modifications, the most frequent being explant with replacement. There were 2 explants due to infection in 2 (1%) subjects, 1 explant due to suicidal ideation in 1 subject, no intracranial hemorrhages, and no deaths related to DBS. During the second year of follow-up, 1 patient had died with definite sudden unexpected death in epilepsy (SUDEP).

### Classification of Evidence

This study provides Class IV evidence that ANT DBS reduces the frequency of seizures in patients with drug-resistant focal epilepsy.

## Discussion

The MORE multinational registry provides systematically collected real-world data about the safety and effectiveness of ANT DBS therapy in patients with DRE. Our results support the clinical efficacy and safety of ANT DBS therapy previously reported in the SANTE randomized controlled trial and single-center experiences. Key findings include a 33.1% median monthly SF reduction after 2 years of treatment; however, it was observed that seizure outcomes of the patients were affected by the treatment center experience. We did not identify new safety concerns regarding the surgical procedure– or treatment-related adverse events compared with the original SANTE trial.^5^

In the subgroup of patients reaching 5 years, the total SR was reported to be 55.1%. Results from this subgroup suggest that there was a continued decrease in SF beyond 2 years, which is consistent with SANTE results^6^ and those from other neuromodulation therapies including VNS^25^ and responsive neurostimulation.^26^ This trend is being evaluated with additional exploratory analyses and will be reported on separately.

The median monthly SF at 2 years of follow-up in MORE was 8.8 compared with 15.8 at baseline, representing a 33.1% median reduction across all seizure types. When categorizing the patients into 3 groups, 32% were responders, whereas 29% had no reduction in SF (no benefit). The remainder of patients had a decrease in SF (improvers) without reaching the response threshold of 50%. The SR of MORE at 2 years was not as high as the one in the open-label extension of SANTE, which showed a 56.0% median reduction in SF. In addition, 54% of patients had an SF reduction of at least 50%, whereas 10% had an increase in their seizure count.^5^ On the other hand, the efficacy results of the MORE registry are comparable with real-world data regarding other neurostimulation therapies, such as VNS, in which long-term median SR by 1 year of therapy was less than 30% but increased to more than 50% by 5 years of stimulation.^27^ We have identified 5 different factors with clinically relevant influence on the SR.

First, the presence or absence of cognitive impairment had some effect on seizure outcome in MORE without reaching statistical significance. Indeed, patients with cognitive impairment had a 26.0% median monthly SR by 2 years compared with 36.1% reduction in those without cognitive impairment. Conversely, SANTE excluded patients with an IQ of less than 70.^5^ There are some data providing support for the significance of cognitive impairment on seizure outcomes. Patients with cognitive impairment, especially regarding executive functions, were less likely to be responders to ANT DBS therapy in a single-center study.^28^ Similar findings were published concerning VNS in a pediatric population.^29^ These results of MORE suggest that patients without cognitive impairment may be better candidates for ANT DBS treatment, but this issue needs to be addressed in further studies.

Second, the region of the brain where the seizure most likely originates may be relevant for seizure outcomes. In the MORE registry, the responder group included numerically more patients with temporal lobe epilepsy compared with the no-benefit group. Only 39% of patients in MORE had temporal lobe epilepsy compared with 60% in SANTE, whereas the proportion of patients with frontal lobe, parietal lobe, or occipital lobe epilepsy was quite similar in both studies. Furthermore, in MORE, the patients with no benefit were more often found to have multifocal epilepsy than the responders, suggesting that those patients may be less likely to respond to ANT DBS therapy. Remarkably, only 9% of SANTE trial patients had diffuse or multifocal epilepsy compared with 24% of patients in MORE. In SANTE, by 2 years, patients with seizures of temporal origin achieved relatively greater benefit of stimulation compared with those with seizures originating from other lobes or multifocal in origin.^5^

Third, the distribution of seizure types and frequencies may have an effect on SR. The baseline median seizure frequencies were similar in MORE and SANTE, but in MORE, there was more variation in SF, which may have contributed to seizure outcomes in MORE. There was also more variation in MORE regarding seizure types, which may also reflect the inherent difficulty in seizure classification in real life.

Fourth, in MORE, the median number of failed ASMs was 9, suggesting a very refractory group of patients with epilepsy. In SANTE, the number of failed ASMs had to be at least 3, but the total number of ASMs was not reported.^5^ In MORE, patients had a median number of 3 concomitant ASMs compared with 2 ASMs in SANTE. High ASM burden usually suggests more refractory epilepsy, which again may have an effect on seizure outcomes. On the other hand, the previous and present surgical treatment modalities (resective surgery and VNS) were similar in MORE and SANTE, as well as the duration of epilepsy and the age at implantation. To avoid cognitive,^30^ psychiatric,^31^ and other ASM-related tolerability effects, ANT DBS or other surgical therapies should probably be considered before adding the third or fourth ASM in patients with DRE.

Finally, in MORE, centers with over 10 implantations showed significantly better seizure outcomes compared with those with 10 or less implantations: 42.8% median monthly SR compared with 25.8%, respectively. Several factors may affect the greater outcome variability among the centers in MORE compared with that of SANTE. The surgical approach in MORE included both transventricular and extraventricular approaches, with 2 different lead models (3387 and 3389), whereas in SANTE, only the transventricular technique and lead model 3387 were used.^5^ In an early surgical analysis of the MORE registry, implantation using the extraventricular approach resulted in a different target location in the ANT area when compared with the transventricular approach.^32^ Conversely, there was a centralized evaluation of targeting in SANTE.^5^ The issues of implantation route or contact location and volume of tissue activated will be analyzed in detail in a separate paper on surgical aspects of MORE. On the other hand, for most patients, stimulation protocols did not differ between MORE and SANTE, although in MORE, requirements regarding the stimulation parameters were not specified. The MORE registry results suggest that the implantation center should be dedicated to optimization of the implantation protocol to reach better efficacy results.

Real-world epilepsy studies such as the MORE registry reliably provide safety-related information because the precision of tolerability reporting is more likely to be accurate than effectiveness-related data. This is of utmost importance because randomized and controlled studies have limitations regarding the inclusion of patients.^5^

In MORE, the rates of depression and memory complaints were in line with those reported in SANTE for the first 2 years of follow-up. In MORE, 13% of patients reported depression during the entire 2-year follow-up period compared with 15% of patients in the active group of SANTE during the 3-month double-blind period.^5^ In patients with BDI measurements, we did not observe any significant differences in BDI scores during active stimulation compared with the baseline. The proportion of patients reporting memory impairment of MORE was 15% during the whole 2-year follow-up compared with 13% of SANTE patients with active stimulation during the double-blind phase.^5^

Serious complications leading to discontinuation of the therapy were rare among patients in MORE confirming the safety data from SANTE. Indeed, none of the patients in the MORE registry discontinued the therapy during the first year after the implantation. Conversely, in SANTE, 5 patients discontinued the therapy during the initial 13-month open-label phase.^5^ During the second year of follow-up, 1 patient had died in MORE of confirmed SUDEP, 3 patients were not available for follow-up (1 was lost for follow-up, and in 2 patients, the withdrawal decision was made by the treating physician without specified cause), and 2 additional patients were withdrawn potentially due to infections. During the corresponding time period, 3 SANTE patients discontinued the therapy.^5^

The HRQOL measurements in MORE did not reach the minimally important clinical difference threshold in QOLIE-31. This finding was not unexpected because several studies suggest that the achievement of seizure freedom in patients with epilepsy, rather than any reduction in SF, significantly affects the HRQOL scales.^33^ This may reflect the lack of sensitivity of quality of life measures to clinically relevant but nontotal reductions in SF. According to a recent Delphi consensus statement, the physicians preferred to rely more on unstructured interviews than formal questionnaires when assessing the effect of ANT DBS therapy on HRQOL.^22^

The strengths of this study include a comprehensive and systematic data collection in a multinational multicenter setting enabling a practical and clinically relevant approach to explore patient characteristics, the surgical procedure, and both effectiveness and safety aspects. The MORE registry provides a real-world adjunct to the results of the SANTE randomized trial. Limitations include reduced reliability of the seizure diaries, nonprotocolized visit windows, and optional questionnaires (HRQOL and BDI performed only in a subset of patients), chosen to respect clinical practice. Finally, some patients were included in the MORE registry retrospectively rather than prospectively, a fact that may have resulted in selection bias.

ANT DBS therapy is evolving as new versions of DBS devices are being developed. In the SANTE trial, the DBS model Kinetra (Medtronic) was used, whereas in the MORE registry, patients were implanted with the Activa PC. Recently, a new neurostimulator was introduced, which offers a recording of local field potentials. This may offer a possibility for better understanding the therapy and treatment optimization in patients with DRE.

In conclusion, the large real-world MORE registry strengthens the evidence supporting the safety of ANT DBS therapy. We confirmed the effectiveness of the ANT DBS treatment and obtained important data suggesting cognitive impairment and region of the brain where the seizure most likely originates as possible features for optimal patient selection. In addition, ANT DBS should be considered in patients with DRE before increasing the ASM burden to avoid a negative effect in terms of side effects. The centers performing ANT DBS implantations should have a long-term comprehensive commitment to patient selection, implantation, and therapy optimization.

## Glossary

AE: adverse event
ANT DBS: DBS of the anterior nucleus of the thalamus
APS: all patients set
ASM: antiseizure medication
BDI-II: Beck Depression Inventory II
DBS: deep brain stimulation
DRE: drug-resistant epilepsy
FAS: full analysis set
FBTCS: focal to bilateral tonic-clonic seizure
FIAS: focal impaired awareness seizure
HRQOL: health-related quality of life
ILAE: International League Against Epilepsy
ITT: intent to treat
MORE: Medtronic Registry for Epilepsy
QOLIE-31: Quality of Life in Epilepsy 31-item questionnaire
RR: responder rate
SAE: serious AE
SANTE: Stimulation of the Anterior Nucleus of the Thalamus for Epilepsy
SF: seizure frequency
SR: seizure reduction
SUDEP: sudden unexpected death in epilepsy
VNS: vagus nerve stimulation
